# Screening and Evaluation of Chassis Cells for Heterologous Biosynthesis of Gas Vesicles as Ultrasound Contrast Agents

**DOI:** 10.3390/md24030106

**Published:** 2026-03-11

**Authors:** Qiuxia Fu, Kezhi Yu, Yuanyuan Wang, Chenxing Liu, Wei Liu, Wenze Ou, Wei Sun, Fei Yan

**Affiliations:** 1 Department of Biomedical Engineering, Shenyang Pharmaceutical University, Shenyang 110016, China; 2 State Key Laboratory of Quantitative Synthetic Biology, Shenzhen Institute of Synthetic Biology, Shenzhen Institutes of Advanced Technology, Chinese Academy of Sciences, Shenzhen 518055, China; 3 Department of Ultrasound, Fifth Affiliated Hospital of Sun Yat-sen University, Zhuhai 519000, China

**Keywords:** gas vesicles, chassis cells, heterologous expression, ultrasound imaging

## Abstract

Gas vesicles (GVs) are hollow protein nanostructures derived from microorganisms and show significant potential for ultrasound imaging applications. However, the direct production of gas vesicles (GVs) from their native hosts faces several limitations: poor contrast imaging performance, insufficient yield, and high costs associated with extraction and purification. These challenges heavily hinder their clinical translation and application. The heterologous expression of GV genes varies significantly among different chassis strains due to their distinct intracellular environments, which ultimately affects GV performance and yield. Therefore, it is crucial to select an appropriate chassis cell that can produce GVs with excellent imaging performance. In this study, the GV gene cluster from *Serratia* sp. ATCC 39006 was heterologously expressed in five different bacterial chassis strains: *Escherichia coli* BL21 (AI), *Escherichia coli* K-12 MG1655, *Escherichia coli* Nissle 1917, *Salmonella* YB1, and *Vibrio natriegens*. By systematically comparing the yield, particle morphology, and ultrasound imaging performance of GVs produced by these strains, we elucidated the impact of chassis cells on GV synthesis and function. This work provides experimental evidence and theoretical support for screening robust GV-producing strains and facilitates future biomedical applications of GVs.

## 1. Introduction

Ultrasound imaging is widely used in clinical diagnosis due to its non-ionizing, non-invasive, and real-time capabilities, along with low cost and ease of operation [1,2,3]. Ultrasound contrast agents can significantly enhance tissue echo signals, improving imaging sensitivity and contrast [4,5]. However, conventional microbubble-based agents (1–10 µm in diameter) are confined to the vascular lumen due to their microscale size and suffer from a short half-life in circulation [6,7]. Moreover, chemically synthesized nanoscale contrast agents often encounter challenges such as complex preparation processes, suboptimal biocompatibility, and heterogeneous particle size distribution [8,9]. In recent years, nanoscale ultrasound contrast agents have attracted increasing attention for overcoming these shortcomings.

Gas vesicles (GVs) are hollow protein nanostructures naturally produced by microorganisms such as cyanobacteria and halophilic archaea [10,11,12]. Studies have shown that GVs possess favorable acoustic scattering properties, excellent biocompatibility, low toxicity, and uniform structure, making them promising candidates as novel acoustic contrast agents [13,14,15]. Their contrast imaging effectiveness has been validated in various animal models [16,17,18]. Nonetheless, direct production of GVs from native hosts is hampered by their suboptimal imaging contrast, low yield, and high purification costs [19,20,21]. Genetically engineered technology provides an ideal platform for constructing microbial cell factories for heterologous GV production, thanks to their well-characterized genetic backgrounds and mature genetic toolkits [22,23,24].

It should be noted that heterologous gene expression can significantly vary among different chassis strains due to their distinct intracellular environments, which may in turn affect both the yield and properties of GVs. Therefore, it is crucial to select an appropriate chassis cell that can produce GVs with excellent imaging performance. In this study, the five typical chassis strains were selected on the basis on the following considerations: *Escherichia coli* BL21 (AI) is a well-established standard industrial strain for high-level recombinant protein expression [25,26]; *Escherichia coli* K-12 MG1655 is a prototypic laboratory strain with a well-defined genetic background [27]; *Escherichia coli* Nissle 1917 (EcN) is a probiotic strain with demonstrated safety and therapeutic potential [28,29]; *Salmonella* YB1 is an attenuated tumor-targeting strain [30,31]; and *Vibrio natriegens* is a fast-growing marine bacterium with high protein synthesis capacity [32,33]. The objective of this study is to systematically evaluate the influence of chassis cells on the synthesis of GVs from *Serratia* sp. ATCC 39006 in five chassis strains, including *E*. *coli* BL21 (AI), *E. coli* K-12 MG1655, *E*. *coli* Nissle 1917, *Salmonella* YB1, and *Vibrio natriegens*, followed by comprehensive comparisons of their yield, morphology, and ultrasound imaging performance of these heterologously synthesized GVs from these different chassis cells.

**Figure 1:**
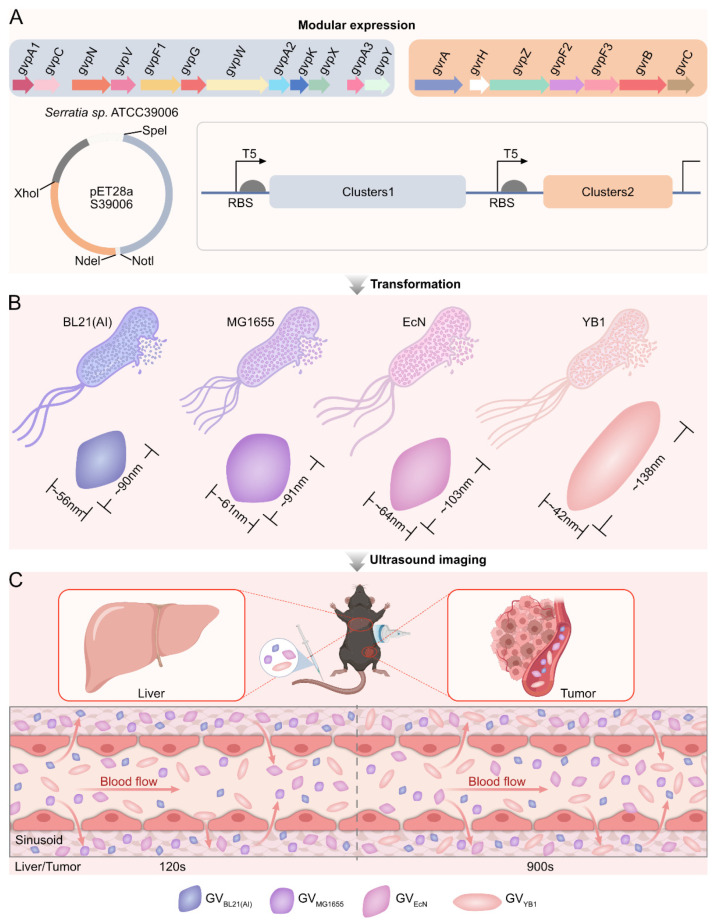
Heterologous expression and *in vivo* imaging of GVs. (**A**) Schematic of GV expression plasmid construction. The GV gene cluster from *Serratia* sp. ATCC 39006 was split into two subclusters and cloned into the pET28a vector to generate a complete functional unit for heterologous expression. (**B**) Expression of GVs in different chassis cells. The verified recombinant plasmid was transformed into five different strains for heterologous expression of GVs. (**C**) *In vivo* ultrasound imaging of GVs. Purified GVs were systemically administered to both healthy mice and tumor-bearing mice, with subsequent imaging of the liver and tumor to assess their ultrasound imaging performance.

In this study, the GV gene cluster from *Serratia* sp. ATCC 39006 was split into two subclusters and cloned into the pET28a vector to generate a complete functional unit for heterologous expression (Figure 1A). The verified recombinant plasmid was then transformed into five different chassis strains for heterologous expression of GVs (Figure 1B). Subsequently, purified GVs were systemically administered to both healthy mice and tumor-bearing mice, with subsequent imaging of the liver and tumor to assess their ultrasound imaging performance (Figure 1C).

## 2. Results

### 2.1. Synthesis of GVs in Different Chassis Cells

**Figure 2:**
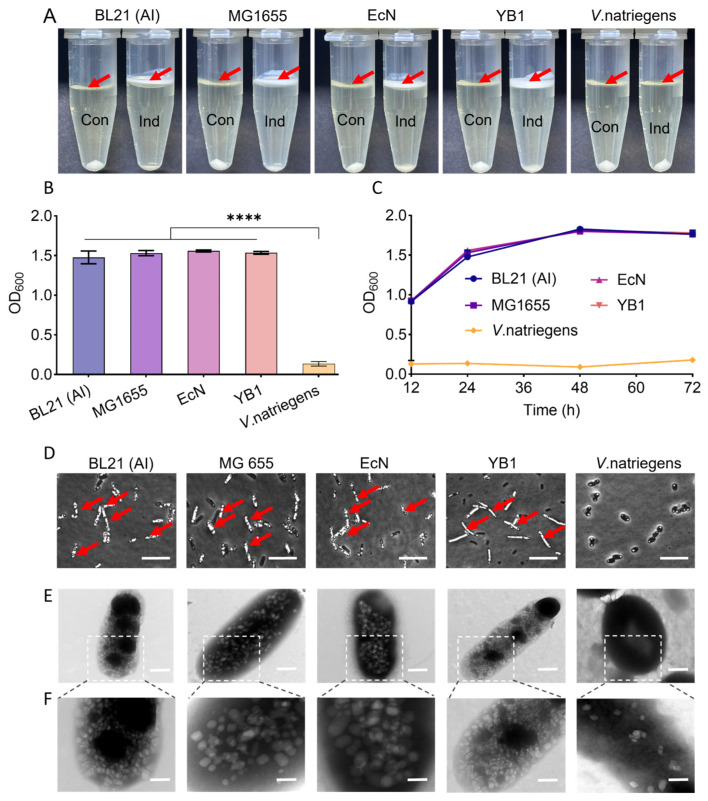
Bacterial synthesis and characterization of GVs. (**A**) Five engineered chassis strains after centrifugation. Arrows point to buoyant, GV-containing cells. (**B**) OD_600_ measurement of buoyant fractions after 24 h of enrichment culture. (**C**) OD_600_ dynamics of buoyant fractions over a 12–72 h culture period. (**D**) Phase-contrast microscopy (PCM) images showing GV synthesis. Arrows indicate intracellular GVs. Scale bar: 10 µm; (**E**,**F**) Scanning electron microscopy (SEM) images of five chassis strains producing GVs (**E**) and magnified view of the boxed area in E (**F**); scale bars: 400 nm (**E**) and 200 nm (**F**); **** *p* < 0.0001.

The modular engineering plasmid was successfully constructed and transformed into different chassis cells, including *E. coli* BL21 (AI), *E. coli* K-12 MG1655, *E. coli* Nissle 1917 (EcN), *Salmonella* YB1, and *Vibrio natriegens*. The yield of GVs was initially assessed by measuring the volume of floating bacterial cells after centrifugation. As shown in Figure 2A, except for *Vibrio natriegens*, which did not show obvious floating bacterial cells, all four other floating cells could be seen on the top of the media, indicative of significant GVs synthesis. Moreover, the addition of IPTG further significantly increased the yield of GVs. Subsequently, we systematically evaluated the GV synthesis dynamics in different chassis cells through quantitative measurement of the floating bacterial amount. As shown in Figure 2B, all four kinds of chassis cells except for *Vibrio natriegens* demonstrated effective GV synthesis with comparable production efficiency after 24 h of IPTG induction (*p* < 0.05). Furthermore, we conducted quantitative tracking of the GV production process across all five chassis cells at different time points. Figure 2C showed that GV yield progressively increased over time in *E. coli*, BL21 (AI), MG1655, EcN, and *Salmonella* YB1 within 48 h after inducer addition. Phase-contrast microscopy clearly revealed that GVs appeared within *E. coli* BL21 (AI), MG1655, EcN, and *Salmonella* YB1, but not in *Vibrio natriegens* (Figure 2D and Figure S1). Transmission electron microscopy (TEM) further confirmed the presence of GVs in the four kinds of strains, while *Vibrio natriegens* produced significantly fewer GVs (Figure 2E,F). Collectively, these results demonstrate that *E. coli* BL21 (AI), MG1655, EcN, and *Salmonella* YB1 effectively produced GVs and are suitable chassis cells for heterologous synthesis of GVs.

### 2.2. Extraction and Characterization of GVs

**Figure 3:**
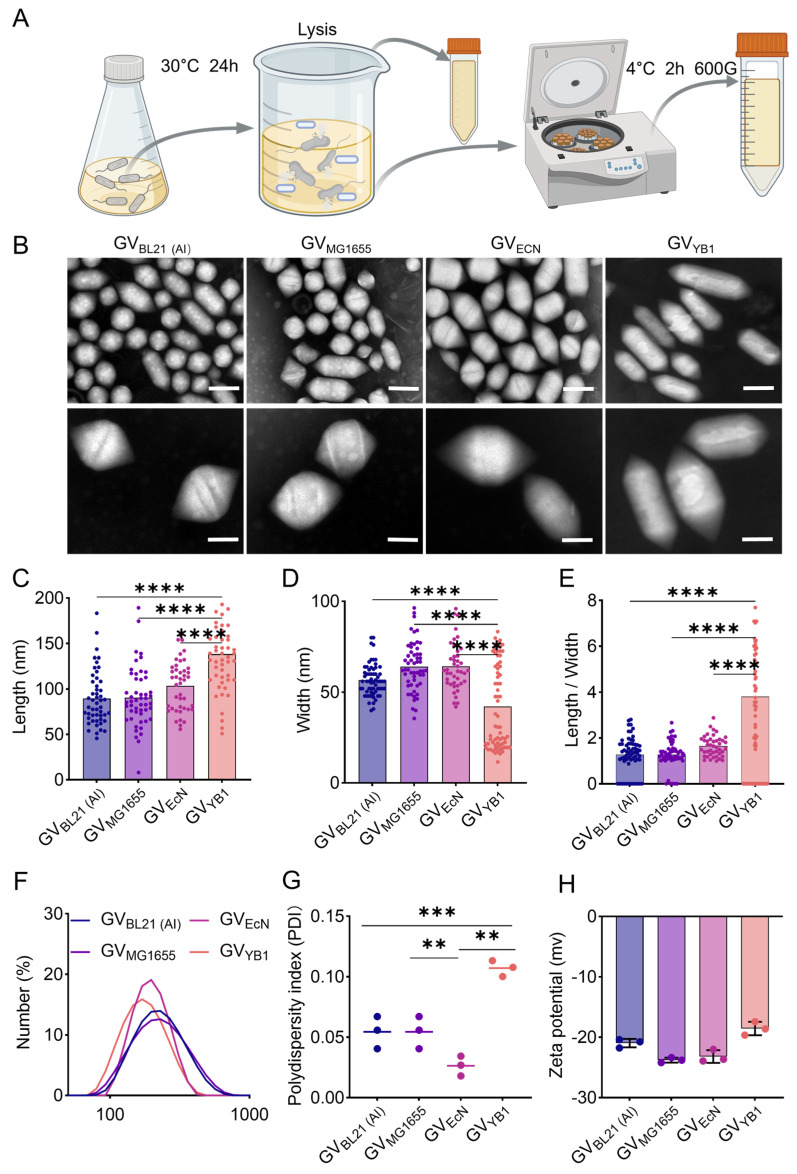
Extraction and characterization of GVs. (**A**) Schematic of the biosynthetic GV production. (**B**) TEM images of GVs isolated from four chassis strains: BL21 (AI), MG1655, EcN1917, and YB1. Scale bars: 200 nm (top row) and 100 nm (bottom row). (**C**–**E**) Quantification of GV dimensions: Length (**C**), width (**D**), and length/width ratio (**E**) derived from the four kinds of strains (*n* = 50 GVs per strain). (**F**–**H**) Hydrodynamic size distribution (**F**), polydispersity index (PDI, (**G**)), and zeta potential (**H**) of the four kinds of GVs. Data are presented as mean ± SD. ** *p* < 0.01, *** *p* < 0.001 and **** *p* < 0.0001.

The GVs were extracted and purified from *E. coli* BL21 (AI), MG1655, EcN, as well as *Salmonella* YB1 according to a previously established protocol (Figure 3A) [34]. TEM imaging revealed that the purified gas vesicles from BL21 (AI) and MG1655 strains exhibited a consistent cylindrical morphology, whereas those from the EcN strain presented a distinct, elongated rod-shaped structure (Figure 3B). Next, we measured the dimensions under TEM, which showed that the mean length of GV_BL21 (AI)_, GV_MG1655_, GV _EcN1917_ and GV_YB1_ were 89.54 ± 29.7 nm, 90.61 ± 32.51 nm, 103.60 ± 27.69 nm, and 138.2 ± 44.8 nm, respectively (Figure 3C), and the width of GV_BL21 (AI)_, GV_MG1655_, GV _EcN_, and GV_YB1_ were 56.77 ± 8.83 nm, 64.23 ± 13.34 nm, 64.27 ± 12.99 nm, and 42.24 ± 23.25 nm, respectively (Figure 3D). The length/width ratios of GVs varied across different chassis cells, as shown in Figure 3E. Briefly, the aspect ratios for GV_BL21 (AI)_, GV_MG1655_, GV_EcN_, and GV_YB1_ were 1.27 ± 0.79, 1.27 ± 0.58, 1.64 ± 0.44, and 3.80 ± 3.31, respectively.

Also, we measured their hydrodynamic diameter in hydration using dynamic light scattering (DLS). The results showed that the hydrodynamic diameter of GV_BL21 (AI)_, GV_MG1655_, GV_EcN_, and GV_YB1_ were 184.2 ± 3.12 nm, 151.4 ± 2.61 nm, 147.3 ± 1.62 nm, and 217.7 ± 11.59 nm, respectively (Figure 3F). The polydispersity index (PDI) measurements indicated good homogeneity across all four kinds of GVs, with the values of 0.05 ± 0.01, 0.05 ± 0.01, 0.03 ± 0.01, and 0.11 ± 0.01 for GV_BL21 (AI)_, GV_MG1655_, GV_EcN_, and GV_YB1_, respectively (Figure 3G). Furthermore, the zeta potentials of GV_BL21 (AI)_, GV_MG1655_, GV_EcN_, and GV_YB1_ were measured at −20.96 ± 0.71 mV, −23.77 ± 0.41 mV, 23.18 ± 1.04 mV, and −18.55 ± 1.12 mV, respectively (Figure 3H). Thus, these results demonstrate that GVs had a uniform nanoscale particle size.

### 2.3. In Vitro Ultrasound Imaging and Stability Assessment of GVs

**Figure 4:**
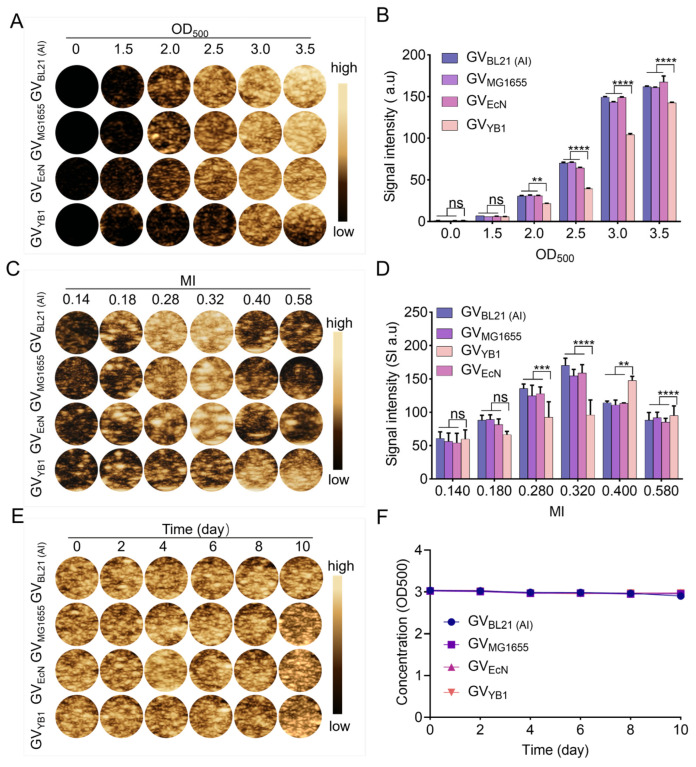
Ultrasound contrast imaging of GVs *in vitro*. (**A**,**B**) *In vitro* ultrasound contrast imaging of GVs at equivalent concentrations (OD_500_ 0–3.5) and their quantification analysis (**B**); (**C**,**D**) representative contrast images (**C**) and the corresponding quantitative analysis of signal intensity (**D**) for GVs at OD_500_ = 3.3 under different mechanical index (MI) conditions; (**E**,**F**) representative contrast images (**E**) and quantitative analysis of signal intensity (**F**) of GVs at OD_500_ = 3.3 at the indicated time points. Data represent the mean ± SD, ** *p* < 0.01, *** *p* < 0.001, and **** *p* < 0.0001, ns, not significant.

Next, we assessed the ultrasound contrast imaging performance of GVs in agar phantoms using a clinical ultrasound system equipped with a 7.1 MHz linear array transducer *in vitro*. The imaging signal intensity showed a positive correlation with GV concentrations across an OD_500_ range of 0 to 3.5 (Figure 4A,B). Notably, for GV_YB1_ at OD_500_ 3.5, significant acoustic attenuation occurred, resulting in a diminished signal in the bottom region of the gel well (Figure 4A). Based on these results, we set the experimental concentration at OD_500_ = 3.3. At this concentration, all four kinds of GVs exhibited favorable ultrasound imaging signal intensities and effectively avoided the signal attenuation observed at the highest concentration (OD_500_ = 3.5) (Figure S2).

Subsequently, at this experimental concentration, we investigated the ultrasound imaging performance of GVs under various mechanical indices (MI). As shown in Figure 4C,D, the average ultrasound signal intensities within the region of interest (ROI) for all four kinds of GVs increased with the mechanical index (MI) until reaching their respective optima. Specifically, GV_BL21 (AI)_, GV_MG1655_, and GV_EcN1917_ peaked at MI = 0.32, with signal intensities of 170.3 ± 10.82 a.u., 154.6 ± 9.65 a.u., and 159.0 ± 12.50 a.u., respectively. In contrast, GV_YB1_ achieved its optimal imaging at a higher MI of 0.40, with an intensity of 147.8 ± 6.12 a.u. Beyond these optimal MI values, further increases in acoustic power led to a decline in signal intensity for all GVs. This phenomenon is likely due to the acoustic collapse of GVs when the acoustic power exceeds their optimal imaging threshold.

To further assess their imaging stability, the four kinds of GVs (OD_500_ = 3.3) were stored at 4 °C and imaged at regular intervals over 10 days. The results showed no significant decrease in contrast signal intensity throughout the observation period (Figure 4E,F). These results demonstrate that GVs produced from these four different chassis cells exhibit favorable imaging stability, establishing a foundation for their further biomedical applications.

### 2.4. In Vivo Ultrasound Imaging of GVs in Normal Liver Tissue

**Figure 5:**
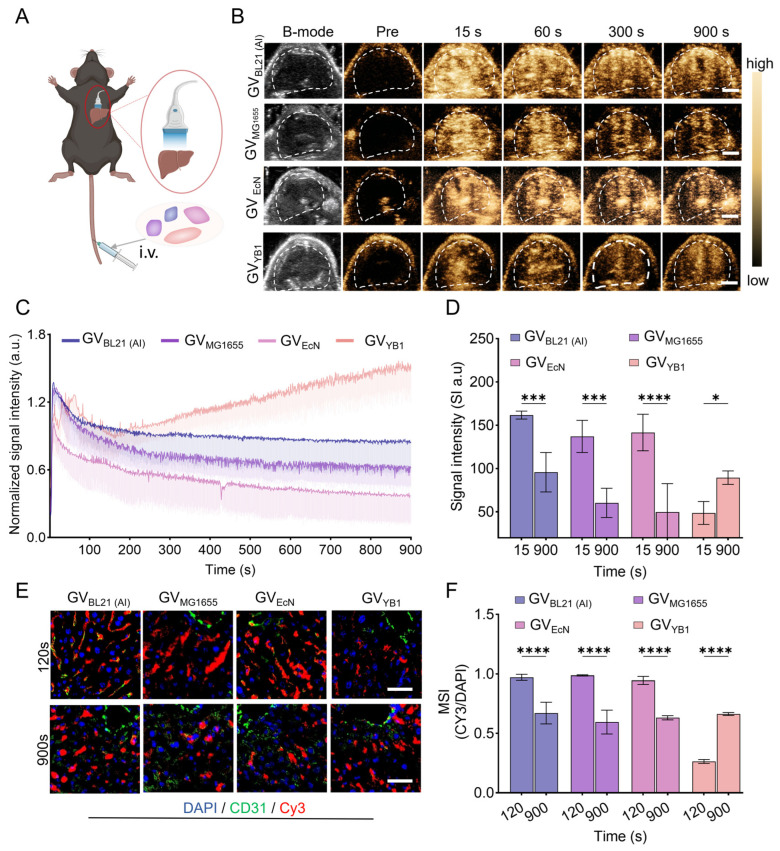
*In vivo* imaging performance of GVs in the liver. (**A**) Schematic of liver imaging in healthy mice for GV_BL21 (AI)_, GV_MG1655_, GV_EcN_, and GV_YB1_; (**B**) *in vivo* hepatic contrast-enhanced ultrasound images at different time points after systemic injection of the four kinds of GVs (*n* = 3). Scale bar: 5 µm; (**C**) normalized time-signal intensity curve for contrast enhancement in (**B**); (**D**) quantitative analysis of liver imaging signal intensity for the four kinds of GVs at 15 s and 900 s; (**E**) immunofluorescence images of the mouse liver at 120 s and 900 s. Cy3-labeled GVs (red); CD31-stained vessels (green); DAPI-stained nuclei (blue). *n* = 3 and scale bar: 40 nm; (**F**) quantitative analysis of average immunofluorescence intensity (CY3/DAPI) in (**E**). Data in (**E**,**F**) are represented as mean ± SD. * *p* < 0.05; *** *p* < 0.001; and **** *p* < 0.0001.

Given their outstanding stability and imaging performance *in vitro*, the four kinds of GVs produced from distinct chassis cells were further compared for their liver imaging capability in healthy C57BL/6J mice. The experimental procedure was illustrated in Figure 5A. Following standardization to OD_500_ = 3.3, each of the four kinds of GVs was systemically injected into mice at a dose of 200 µL. Figure 5B,C showed that the kinetics of contrast-enhanced ultrasound (CEUS) imaging varied significantly across the different GV types. Specifically, GV_BL21 (AI)_, GV_MG1655_, and GV_EcN_ showed similar kinetics, exhibiting rapid contrast enhancement that peaked at approximately 15 s post-injection, followed by a gradual signal decline over 900 s. In contrast, GV_YB1_ displayed a unique pharmacokinetic profile characterized by progressive signal accumulation, reaching its peak at the 900 s. Quantitative analysis detailed the signal attenuation dynamics over a 900 s period (Figure 5D). Specifically, the ultrasound signal intensity from the peak (approximately 15 s) to 900 s changed as follows: GV_BL21 (AI)_ decreased from 161.70 ± 4.55 a.u. to 95.79 ± 22.75 a.u. (with a reduction of about 15%); GV_MG1655_ decreased from 137.20 ± 21.08 a.u. to 60.35 ± 16.91 a.u. (with a reduction of about 38%); and GV_EcN_ decreased from 141.60 ± 21.08 a.u. to 49.76 ± 32.89 a.u. (with a reduction of about 34%). In contrast, the signal intensity of GV_YB1_ increased from 48.65 ± 13.23 a.u. to 89.48 ± 7.71 a.u., representing an increase of 34% by 900 s. The distinct perfusion imaging performance of GV_YB1_ may be attributed to its elongated rod-shaped structure, which affects its extravasation kinetics in the hepatic sinusoids, leading to prolonged imaging duration and delayed time to peak. The hepatic sinusoids are characterized by endothelial fenestrae and a discontinuous basement membrane, providing the primary pathway for nanoparticle extravasation from the vasculature into the liver tissue. Compared with the more oval-shaped structures of the other three GV variants, the elongated rod-like morphology of GV_YB1_ likely encounters greater steric hindrance and orientational constraints when passing through the sinusoidal fenestrae, thereby significantly reducing its passive extravasation efficiency. This morphological restriction not only delays its effective penetration from the vessels into the interstitial space (resulting in a delayed time to peak), but also makes it less susceptible to rapid washout by sinusoidal blood flow, thus prolonging its imaging persistence in the liver. Therefore, the morphological characteristics of GV_YB1_ may explain both its lower extravasation efficiency and its prolonged imaging kinetics.

To elucidate the differences in time-to-peak imaging among the various GVs, we performed immunofluorescence staining using Cy3-labeled GVs. Analysis of liver sections showed that a substantial amount of Cy3-labeled GV_BL21 (AI)_, GV_MG1655_, and GV_EcN_ extravasated from the vasculature by 120 s post-injection, with a visible decrease in signal by 900 s. In contrast, Cy3-labeled GV_YB1_ showed minimal vascular infiltration at 120 s post-injection. Notably, it exhibited progressive accumulation within the liver by 900 s (Figure 5E). At 120 s, the mean fluorescence intensities (Cy3/DAPI) for GV_BL21 (AI)_, GV_MG1655_, GV_EcN_, and GV_YB1_ were 0.97 ± 0.03, 0.99 ± 0.01, 0.95 ± 0.03, and 0.26 ± 0.02, respectively. By 900 s, the signals for GV_BL21 (AI)_, GV_MG1655_, and GV_EcN_ had declined to 0.67 ± 0.09, 0.59 ± 0.10, and 0.63 ± 0.02, respectively, while that of GV_YB1_ increased to 0.66 ± 0.01. Quantitative immunofluorescence proved our hypothesis: GV_BL21 (AI)_, GV_MG1655_, and GV_EcN_ showed rapid extravasation within the hepatic vasculature after systemic administration, achieving a fast ultrasound contrast peak, and subsequently being cleared from the tissue. In contrast, GV_YB1_ exhibited a progressive accumulation in the liver over time, with slower extravasation and delayed clearance from the bloodstream. Collectively, our data demonstrate that GVs generated from the selected chassis cells all produce strong ultrasound contrast signals in the liver. Importantly, their hepatic perfusion and retention kinetics vary with GV particle shape. This shape-dependent characteristic, particularly the distinct elongated rod-shaped structure of GV_YB1_, enriches *in vivo* GV dynamics that differ from those of conventional ultrasound contrast agents. This feature may offer great potential for application in the diagnosis of liver diseases.

### 2.5. In Vivo CEUS Imaging of GVs in Tumor

**Figure 6:**
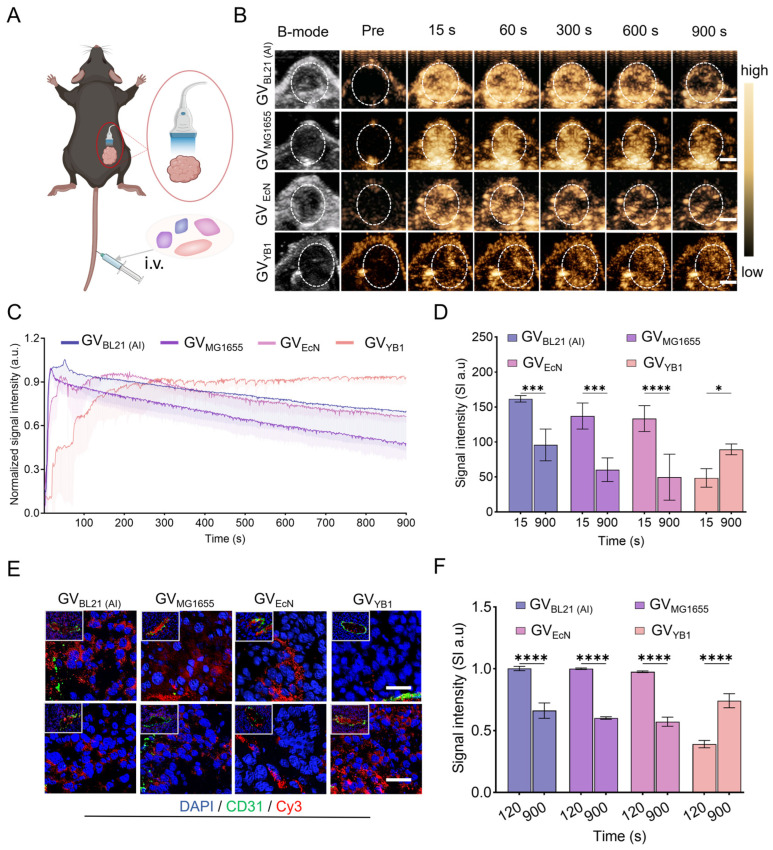
Imaging performance of GVs in tumors. (**A**) Schematic diagram of intratumoral imaging for the four kinds of GVs in mice; (**B**) contrast-enhanced ultrasound images of intratumoral regions at different time points following systemic injection of the four kinds of GVs (*n* = 3). Scale bar: 5 µm; (**C**) normalized time–signal intensity curve for contrast enhancement in (**B**); (**D**) quantitative analysis of tumor imaging signal intensity for the four kinds of GVs at 15 s and 900 s; (**E**) immunofluorescence images of the tumor at 120 s and 900 s. Cy3-labeled GVs (red); CD31-stained vessels (green); DAPI-stained nuclei (blue). *n* = 3, scale bar: 40 nm; (**F**) quantitative analysis of average immunofluorescence intensity (CY3/DAPI) in (**F**). * *p* < 0.05; *** *p* < 0.001; and **** *p* < 0.0001.

To explore the tumor imaging capability of GVs, we intravenously administered GV_BL21 (AI)_, GV_MG1655_, GV_EcN_, and GV_YB1_ at equal concentrations into the tumor-bearing mice, as shown in Figure 6A. From Figure 6B,C, we can see that GV_BL21 (AI)_, GV_MG1655_, GV_EcN_, and GV_YB1_ effectively perfused into the tumors. Consistent with the phenomenon in normal liver, GV_BL21 (AI)_, GV_MG1655_, and GV_EcN_ also exhibited rapid contrast enhancement within tumors, peaking at approximately 15 s post-systemic injection, followed by a gradual signal decay over time. In contrast, GV_YB1_ showed markedly delayed kinetics, with its intratumoral signal progressively increasing after administration. Quantitative analysis of signal intensity (Figure 6D) showed that at 15 s, GV_BL21 (AI)_, GV_MG1655_, and GV_EcN_ reached peak values of 161.7 ± 4.54, 136.9 ± 19.03, and 133.5 ± 18.5 a.u., respectively, whereas GV_YB1_ was significantly lower (55.31 ± 20.93 a.u.). By 900 s, signals from the GV_BL21 (AI)_, GV_MG1655_, and GV_EcN_ declined to 95.79 ± 22.75, 60.35 ± 16.91, and 49.76 ± 49.76 a.u., respectively. In contrast, GV_YB1_ increased substantially to 89.48 ± 7.72 a.u., representing a 45.7% rise from its 15 s baseline.

To elucidate the underlying differential intratumoral perfusion of the various GVs, we performed immunofluorescence staining on tumor tissues. After systemic injection of Cy3-labeled GVs, mice were sacrificed at 120 s and 900 s, and tumors were harvested for analysis. The results demonstrated that all four kinds of GVs could effectively extravasate through tumor vascular gaps and reach the peritumoral cellular area. Notably, a substantial amount of GV_BL21 (AI)_, GV_MG1655_, and GV_EcN_ had penetrated the tumor vessel walls and reached tumor cells at the early time point (120 s). In contrast, only a minimal amount of GV_YB1_ extravasating the vasculature was observed. By 900 s, the fluorescence signals from GV_BL21 (AI)_, GV_MG1655_, and GV_EcN_ had decreased markedly in comparison to their levels at 120 s, whereas the signal from GV_YB1_ progressively increased. Quantitative analysis of the mean fluorescence intensity confirmed this trend: the signals for GV_BL21 (AI)_, GV_MG1655_, and GV_EcN_ dropped from 0.99 ± 0.02, 0.99 ± 0.01, and 0.97 ± 0.01 a.u. at 120 s to 0.68 ± 0.07, 0.60 ± 0.01, and 0.58 ± 0.01 a.u. at 900 s, corresponding to an average reduction of ~36.9%. In contrast, the signal for GV_YB1_ rose from 0.39 ± 0.03 a.u. to 0.74 ± 0.05 a.u. over the 900 s period, with an increase of ~34.8%. These findings further support our hypothesis that the elongated, rod-shaped GV_YB1_ impedes its extravasation across tumor vascular gaps. This not only delays the time to peak imaging enhancement but also results in slower clearance by blood flow, leading to prolonged intratumoral imaging duration. Consequently, the choice of chassis cell determines the GV morphology, which in turn dictates different intratumoral imaging characteristics. In addition, the biocompatibility of GVs produced by the four kinds of chassis strains was evaluated through blood biochemistry, complete blood count, and H&E staining of major organs, which confirmed their favorable safety profile *in vivo* (Figures S3 and S4).

## 3. Conclusions

In summary, this study successfully produced gas vesicles (GVs) with distinct morphologies and imaging kinetics through genetically engineering of multiple chassis cells. The results demonstrate that *Escherichia coli* BL21 (AI), MG1655, EcN, and *Salmonella* YB1 can serve as efficient chassis strains, stably producing GVs with high yield and excellent imaging performance. Furthermore, this work clarifies that the rational selection and engineering of chassis cells can modulate GV morphology, thereby directly influencing their imaging performance. These findings not only establish a clear “chassis-GV morphology-imaging performance” relationship but also provide a solid theoretical and experimental foundation for screening and constructing high-efficiency GV-producing strains. Compared with traditional microbubble-based contrast agents, the nanoscale biosynthesized GVs may offer unique advantages such as extravasation from blood vessels, making it possible to achieve molecularly targeted imaging of tumor cells.

## 4. Materials and Methods

### 4.1. Plasmid Construction

The *Serratia* sp. ATCC 39006 strain used in this study was purchased from the American Type Culture Collection (ATCC). Its wild-type genome has a 19-gene gas vesicle (GV) cluster. In this study, the complete 19-gene cluster was divided into two sub-clusters (cluster 1, gvpA-gvpY and cluster 2, gvrA-gvrC) [35,36]. Each sub-cluster was cloned into the downstream of a constitutive T5 promoter for expression by restriction enzyme and ligation. After sequencing verification, the correct assembly of the recombinant plasmid was transformed into different chassis cells for further functional assays. The sequences of primers used in this article are listed in Supplementary Table S1.

### 4.2. Cultivation of GV-Producing Bacteria

The recombinant plasmid encoding gas vesicles (GVs) was transformed into five chassis strains: *Escherichia* coli BL21 (AI), MG1655, EcN, Salmonella YB1, and *Vibrio natriegens*. Positive single colonies were selected and inoculated into 5 mL of LB liquid medium supplemented with 50 μg/mL kanamycin and 0.2% glucose, followed by incubation at 37 °C with shaking for 16 h to prepare seed cultures. Subsequently, the seed cultures at a 1:100 ratios, were transferred into fresh LB medium containing the same concentrations of antibiotic and glucose for continued shaking cultivation. When the cultures’ optical density at 600 nm (OD_600_) reached 0.6–0.7, isopropylβ-D-1-thiogalactopyranoside (IPTG) was added, and the temperature was shifted to 30 °C for 24 h to induce GV synthesis. After induction, to visually assess GV formation, aliquots of culture (1 mL) of each culture were centrifuged at 600× *g* for 10 min. A white suspended layer appeared in the upper phase, indicating the presence of GV-containing bacteria [19,37,38].

### 4.3. Extraction and Purification of GVs

After cultivation of the GV-containing bacteria, the culture was centrifuged at 600× *g* and 4 °C for 2 h to collect the GV-containing bacteria from the upper layer. The harvested cells were gently resuspended in a bacterial active protein extraction reagent (L200500, Galantis, Shenzhen, China) supplemented with lysozyme (L8120, Soleibao, Beijing, China), followed by incubation with gentle stirring at room temperature for 2 h. DNase I (9003-98-9, GLPBIO, Shanghai, China) was then added, and the mixture was incubated for an additional 2 h. After enzymatic digestion, the sample was centrifuged again under the same conditions (600× *g*, 4 °C, 2 h) to obtain a crude GVs [34]. The pellet was subsequently washed three to four times with PBS buffer (2.0 mM KH2PO4, 137 mM NaCL, 10.0 mM NaHPO4, 2.7 mM KCI) (G4202-500ML) (Servicebio, Wuhan, China) to yield purified GVs.

### 4.4. Characterization of GVs

The morphology of GVs was examined by a transmission electron microscope (Hitachi H-7650, Tokyo, Japan) and phase-contrast microscopy (Olympus IX83 inverted microscope, Tokyo, Japan). The particle size and zeta potential of GVs were measured using a particle size analyzer (Malvern Panalytical ZS XPLORER, Malvern, UK).

### 4.5. In Vitro Imaging and Stability Test

In this study, we first explored the effect of GV concentration on ultrasound contrast enhancement. GV solutions with optical densities at OD_500_: 0–3.5 were loaded into pre-fabricated agarose phantom wells. Ultrasound imaging was conducted using a clinical scanner (Resona 9, Mindray, Shenzhen, China) equipped with a linear array probe (L11-3U) [17]. Contrast-enhanced images were acquired at each concentration, followed by quantitative analysis of the signal intensity. The imaging parameters were set as follows: frequency 7.1 MHz, frame rate 10 Hz, dynamic range 115 dB. Following determination of the optimal GV concentration (OD_500_ = 3.3), the mechanical index (MI) was subsequently optimized. Keeping all other imaging parameters constant, the MI was systematically varied from 0.145 to 0.581 to evaluate its effect on GV-mediated contrast enhancement and to identify the optimal imaging conditions [34]. To assess the stability of GVs, samples (OD_500_ = 3.3) were stored at 4 °C, and *in vitro* images were obtained every other day for 10 days [39]. The contrast signal intensities were quantified at each time point.

### 4.6. Cell Culture

B16F10 cells (TCM36, Cell Bank of the Chinese Academy of Sciences, Shanghai, China) were cultured in RPMI-1640 medium (G4535) (Servicebio, Wuhan, China) supplemented with 100 U/mL penicillin, 100 µg/mL streptomycin (G4003) (Servicebio, Wuhan, China), and 10% heat-inactivated fetal bovine serum (G8003) (Servicebio, Wuhan, China).

### 4.7. Establishment of Animal Models

B16F10 cells were cultured to the logarithmic growth phase. When the cell confluence reached 80%, cells were detached with 0.25% trypsin, centrifuged, and resuspended in PBS to prepare a single-cell suspension at 2.0 × 10^5^ cells/mL. Subsequently, 0.1 mL of the cell suspension was subcutaneously inoculated into the flank of C57BL/6 mice. Tumor growth was monitored using an ultrasound imaging system (Resona 9, Mindray, Shenzhen, China), and the contrast imaging experiments were initiated when the tumor diameter reached 5–8 mm [40,41].

### 4.8. In Vivo Ultrasound Imaging of GVs

The tumor-bearing mice were anesthetized, fixed in a supine position on a heated pad (37 °C), and an optimal ultrasound view was obtained. To avoid potential cross-interference, each mouse received an injection of a single type of GV. The injection volume was 200 μL at an OD_500_ of 3.3 [17].

### 4.9. Tissue Immunofluorescence Analysis

Briefly, Cy3-labeled GVs (100 µL, OD_500_ = 3.3) were intravenously injected into tumor-bearing or healthy control mice. At designated time points, mice were euthanized, and tumors were excised, cryosectioned, and immunostained with an anti-CD31 antibody (GB113151, Servicebio, China) to label blood vessels [18]. Sections were then imaged using a confocal laser scanning microscope (Nikon A1R, Tokyo, Japan).

### 4.10. Statistical Analyses

Statistical analyses were performed using GraphPad Prism 9.0. Data normality was assessed with the Shapiro–Wilk test. Data are expressed as mean ± standard deviation (SD). Differences between the two groups were evaluated using a two-tailed Student’s *t*-test or the Mann–Whitney test. Comparisons among multiple groups were conducted by one-way analysis of variance (ANOVA). A *p*-value < 0.05 was considered statistically significant.

## Data Availability

The original data presented in the study are included in the article and the Supplementary Material; further inquiries can be directed to the corresponding author.

## Supplementary Materials

The following supporting information can be downloaded at: https://www.mdpi.com/article/10.3390/md24030106/s1, Figure S1: Five chassis cell strains biosynthesized gas vesicles (GVs) without the inducer. scale bars: 10 µm; Figure S2: *In vitro* ultrasound contrast imaging of GVs at a concentration of OD_500_ = 3.3; Figure S3: Biosafety assessment in mice. Representative H&E-stained sections of major organs (heart, liver, spleen, lung, and kidney) collected 7 days post-administration of PBS or GVs. scale bars: 50 µm; Figure S4: (A–K) Hematological detection of liver function (A,B), kidney function (C,D), and blood count (E–H) 3 days after intravenous injection of PBS or GVs in mice. (*n* = 3); Table S1: Primers used in this study.
