# Supplementary material for: Screening and Evaluation of Chassis Cells for Heterologous Biosynthesis of Gas Vesicles as Ultrasound Contrast Agents
Source: Mar Drugs. 2026 Mar 11;24(3):106. doi: 10.3390/md24030106 (PMC13027821; doi:10.3390/md24030106)
Supplement: Supplementary file 1 [file marinedrugs-24-00106-s001.zip › marinedrugs-4157020-supplementary.pdf]

Supplementary data

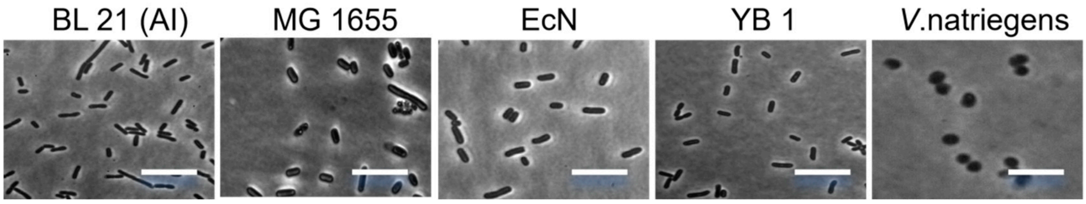

Figure S1: Gas vesicles (GVs) biosynthesized by five kinds of chassis cell strains in the absence of an inducer. Scale bar: 10 μm.

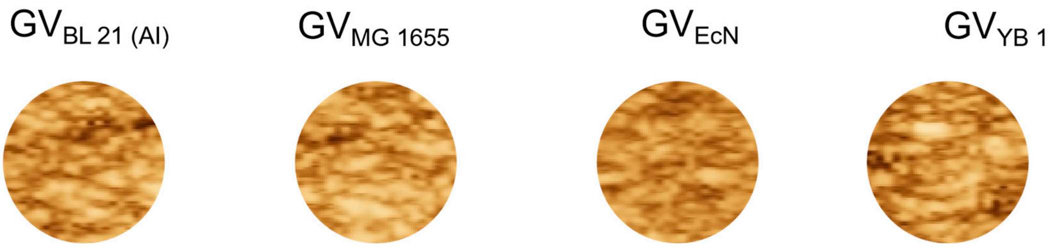

Figure S2. In vitro ultrasound contrast imaging of GVs at a concentration of OD_{500} = 3.3.

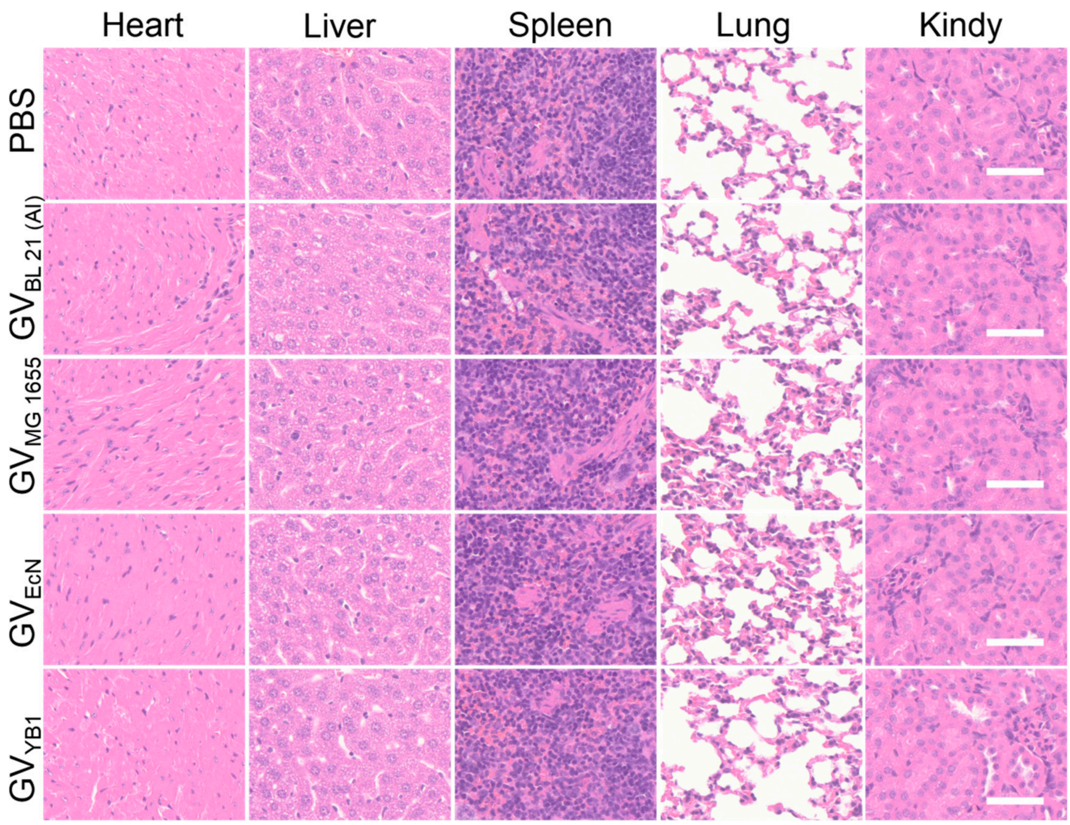

Figure S3. Biosafety assessment in mice. Representative H&E-stained sections of

major organs (heart, liver, spleen, lung, and kidney) collected 7 days post-administration of PBS or GVs. Scale bar: 50 µm.

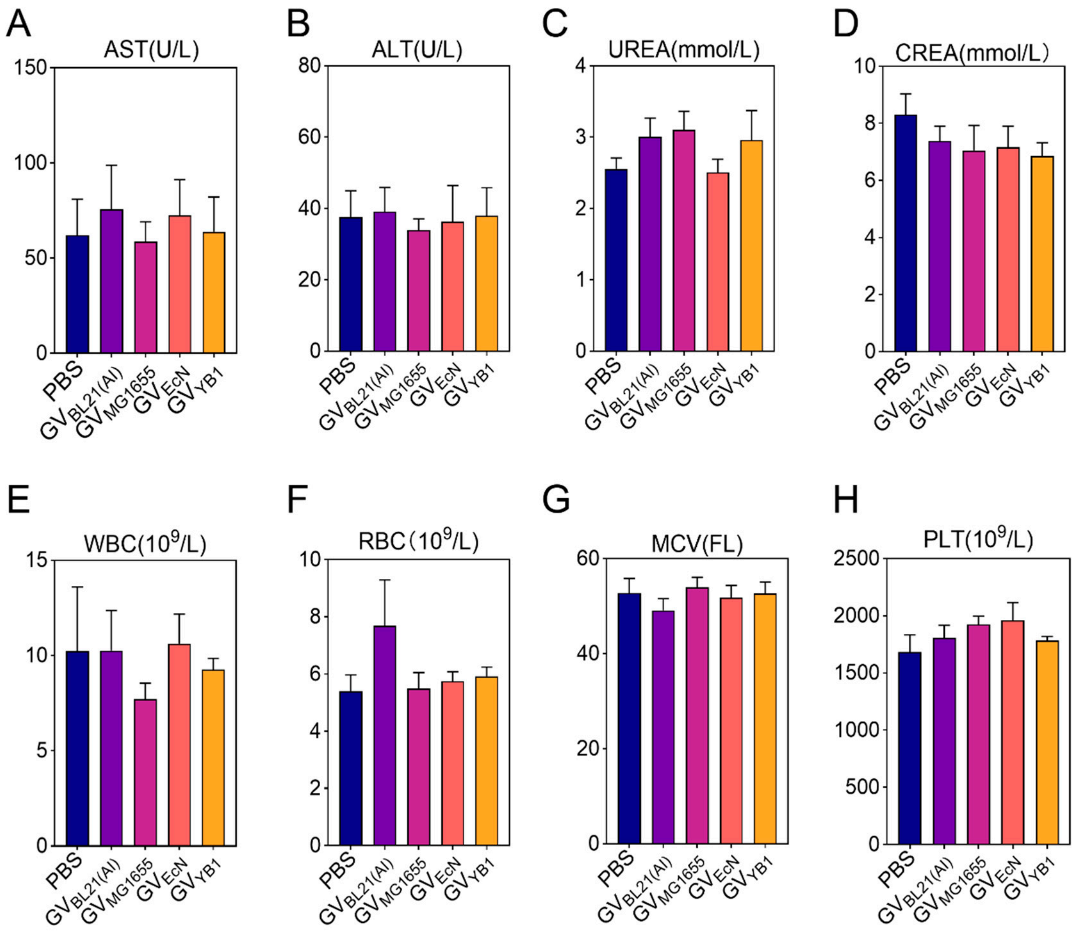

Figures S4. (A-K) Hematological detection of liver function **(A, B)**, kidney function **(C, D)**, and blood count **(E-H)** 3 days after intravenous injection of PBS or GVs in mice. (n = 3).

Table S1. Primers used in this study

Primers	Sequnence (5 to 3)
XhoI-F	CCATCCTATGGAACTGCCTC
XhoI-R	GCCAAGGTACTGGTTTGGGT
NotI-NdeI-F	AACCGAAACTCAAACGCGTG
NdeI-R	AAGTATTGATCCTGGCTATGT
